# Rapid detection of treatment-emergent resistance to bedaquiline and pretomanid in *Mycobacterium tuberculosis* using TM4::*GeNL* reporter mycobacteriophages

**DOI:** 10.1128/spectrum.03900-25

**Published:** 2026-07-15

**Authors:** Senamile L. Ngema, Saranathan Rajagopalan, Rubeshan Perumal, Sashen Moodley, Kevin J. Guzman, Adrie J. C. Steyn, William R. Jacobs, Kogieleum Naidoo, Max O'Donnell, Michelle H. Larsen

**Affiliations:** 1 Centre for the AIDS Programme of Research in South Africa (CAPRISA), University of KwaZulu-Natal 56394, Durban, South Africa; 2 Department of Microbiology and Immunology, Albert Einstein College of Medicine, Bronx, New York, USA; 3 Department of Pulmonology and Critical Care, School of Clinical Medicine, College of Health Sciences, University of KwaZulu-Natal 72753, Durban, South Africa; 4 South African Medical Research Council (SAMRC)–CAPRISA HIV-TB Pathogenesis and Treatment Research Unit, University of KwaZulu-Natal 56394, Durban, South Africa; 5 African Health Research Institute, University of KwaZulu-Natal 56394, Durban, South Africa; 6 Division of Pulmonary, Allergy, and Critical Care Medicine, Columbia University Medical Center 21611, New York, New York, USA; 7 Department of Microbiology, University of Alabama at Birmingham 318277, Birmingham, Alabama, USA; 8 Department of Epidemiology, Columbia University Medical Center 21611, New York, New York, USA; End TB Dx Consulting LLC, San Diego, California, USA

**Keywords:** *Mycobacterium tuberculosis*, bedaquiline, delamanid, pretomanid, drug susceptibility testing, mycobacteriophages

## Abstract

There is an urgent need for rapid, affordable diagnostic tools capable of detecting baseline and treatment-emergent resistance among patients with drug-resistant tuberculosis (DR-TB). Reporter mycobacteriophages provide a phenotypic solution enhanced by brighter luminescent reporters compatible with simple readouts. We applied the TM4::*GeNL* reporter mycobacteriophage for rapid phenotypic drug susceptibility testing (pDST) on serial *Mycobacterium tuberculosis* isolates from six DR-TB patients, including four with treatment-emergent resistance and two with baseline bedaquiline (BDQ)-resistant subpopulations. Susceptibility to BDQ, pretomanid (PMD), delamanid (DLM), and linezolid (LZD) was assessed by luminescence retention after TM4::*GeNL* infection compared to no-drug control, with conventional pDST and whole-genome sequencing as the reference standard. Isolates with baseline and treatment-emergent resistance to BDQ and PMD had a mean relative luminescence unit (RLU) retention of 83.8% (95% CI, 52.1–115.6), while susceptible clinical isolates retained 1.5% mean RLU (95% CI, 0.2–2.8). The TM4::*GeNL* DST results were consistent with conventional pDST in detecting BDQ and PMD resistance. Three patients with BDQ treatment-emergent resistance had mutations in *mmpR5* (*Rv0678*), with a range of variant frequencies (12.6%–92.3%). One isolate with treatment-emergent DLM/PMD resistance had mutations in the *fbiC* gene, with A487fs and S534stop variants at 25.0% and 12.0%, respectively. Sequencing of serial isolates from one patient on a regimen that included LZD identified an emergent mutation in *rrl* (n.2814G > T, 53.8% frequency); however, the isolate was LZD susceptible by both TM4::*GeNL* DST and conventional pDST. TM4::*GeNL* reporter mycobacteriophage accurately detected baseline and treatment-emergent resistance to BDQ and PMD compared to conventional pDST. This rapid, phenotypic approach shows promise for accelerating detection of resistance to new and repurposed DR-TB drugs in clinical settings.

**IMPORTANCE:**

Ongoing transmission of drug-resistant tuberculosis (DR-TB), combined with limited access to rapid diagnostics for resistance to key drugs in modern regimens, poses a major challenge to global TB control. We applied TM4::*GeNL* reporter mycobacteriophage for rapid phenotypic drug susceptibility testing on *Mycobacterium tuberculosis* (*Mtb*) clinical isolates with baseline and treatment-emergent resistance to DR-TB drugs (bedaquiline, pretomanid, and delamanid), generating results within 48 h from a positive culture. Our findings provide evidence on the utility of the TM4::*GeNL* mycobacteriophage to infect *Mtb* clinical isolates and distinguish between drug-susceptible and resistant isolates. TM4::*GeNL* reporter mycobacteriophage detected emerging resistance to bedaquiline and pretomanid within 48 h from a positive culture, with our results correlating well with conventional phenotypic drug susceptibility and whole-genome sequencing data. These findings support TM4::*GeNL* reporter mycobacteriophage as a rapid, multiplexed alternative drug-susceptibility test for DR-TB.

## INTRODUCTION

Drug-resistant tuberculosis (DR-TB) represents a key obstacle to global TB control due to reduced effectiveness of the most potent anti-TB drugs (1). In 2023, among 10.8 million people who fell ill with TB, an estimated 400,000 had multi-drug-resistant or rifampicin-resistant TB (MDR/RR-TB) (2). However, only 189,631 (47%) were microbiologically diagnosed, and only 175,923 (44%) initiated MDR-TB treatment (2). Expanding access to comprehensive resistance testing and improving access to appropriate TB treatment may narrow the diagnosis-treatment gap.

DR-TB treatment has historically been characterized by a high pill burden, prolonged therapy, severe side effects, and poor retention in care, any of which contribute to high rates of treatment failure and mortality (3, 4). However, significant progress has been made in the last decade, with the introduction of novel and repurposed drugs, including bedaquiline (BDQ), pretomanid (PMD), delamanid (DLM), and linezolid (LZD), as part of new all-oral short-course regimens (5–9). The most widely implemented 6–9 month all-oral regimen, comprising BDQ, PMD, LZD, and moxifloxacin (MFX) (BPaL/M), has been associated with reduced side effects and improved treatment outcomes (10–12). The remarkable progress in the introduction of new, highly effective, all-oral DR-TB treatment regimens is threatened by the rise in BDQ resistance, which may undermine recent gains in DR-TB treatment (13–15). The rise in acquired BDQ resistance has been attributed to suboptimal adherence or suboptimal pharmacokinetic-pharmacodynamic parameters in complex TB lesions or disease sites (16–19). Delayed detection of BDQ-resistant *Mycobacterium tuberculosis* (*Mtb*) has been associated with ongoing community transmission of BDQ-resistant TB (20, 21).

Current molecular diagnostics for DR-TB are limited to well-characterized mutations and fail to detect resistance to newer drugs central to modern treatment regimens, rendering them inadequate (22). Resistance mechanisms for BDQ and PMD remain poorly defined, with mutations distributed across multiple genes, complicating an accurate and reliable molecular diagnostic development (23). In addition, there’s a significant gap in our understanding of which mutations in resistance-associated genes lead to phenotypic resistance to new TB drugs. As a result, resistance detection often relies on culture-based phenotypic drug susceptibility testing (pDST), which can take up to 6 weeks, critically delaying treatment decisions. The World Health Organization (WHO) has emphasized the need for simple, rapid, and cost-effective phenotypic approaches to guide timely DR-TB treatment.

Reporter mycobacteriophages have previously been shown to rapidly and accurately determine drug susceptibility to new and repurposed anti-TB drugs, including BDQ, PMD, and LZD (24–27). The recently constructed TM4::*GeNL* mycobacteriophage carrying a green-enhanced nanoluciferase cassette (*GeNL*) has been shown to be ultrasensitive and able to perform DST on all *Mtb* lineage clinical isolates (28).

Early detection of treatment-emergent resistance, coupled with prompt therapeutic decision-making, is critical for providing upfront guidance to inform resistance-appropriate drug selection, limit the transmission of DR-TB strains, and improve outcomes (29). TM4::*GeNL* reporter mycobacteriophage was used for rapid (48 h) phenotypic drug-susceptibility testing of clinical *Mtb* isolates with baseline and treatment-emergent resistance, with results compared to conventional phenotypic and whole-genome sequencing (WGS) data.

## MATERIALS AND METHODS

### *Mycobacterium tuberculosis* clinical isolates

This nested retrospective analysis used stored serial *Mtb* clinical isolates obtained from six participants enrolled in the CAPRISA 086-PRAXIS (ClinicalTrials.gov: NCT03162107), a prospective observational cohort study conducted in Durban, South Africa, to characterize medication adherence and retention in the DR-TB-HIV care continuum among patients receiving BDQ-based regimens. We screened for patients who had persistent positive cultures and had at least a baseline isolate and one or more on-treatment isolates presenting emerging resistance to novel drugs and/or baseline BDQ heteroresistance. We identified six patients, each with between two and six positive culture isolates. All baseline isolates and consecutive isolates were selected to represent a range of genotypic and phenotypic profiles relevant to assessing the performance of the assay. Written informed consent for specimen storage and future use was obtained at enrollment.

Within PRAXIS, extended pDST for BDQ, LZD, and CFZ was performed for isolates exhibiting fluoroquinolone or second-line injectable drug resistance. All isolates underwent comprehensive characterization, including WGS and routine culture-based pDST. BDQ minimum inhibitory concentrations (MICs) were determined for all isolates harboring mutations in *mmpR5, atpE, pepQ*, or *Rv1979c* (15). DLM MICs were determined for all isolates harboring mutations in *ddn*, *fgd1*, *fbiA*, *fbiB*, *fbiC*, or *fbiD*. All clinical isolates were grown in Middlebrook 7H9 medium supplemented with tyloxapol (0.05%) and OADC (10%) and cultured to mid-log phase (OD_600_ ~0.8). All cultures were checked for contamination by inoculating in 5% sheep blood agar plates.

### Phenotypic DST methods

#### Conventional culture-based phenotypic drug susceptibility and MIC testing

All selected clinical isolates had pDST results, which were done routinely as a part of the parental study. Positive cultures were tested for pDST to first-line (rifampicin [1.0 µg/mL], isoniazid [0.2 and 1.0 µg/mL], and ethambutol [7.5 µg/mL]) and second-line (kanamycin [6.0 µg/mL] and ofloxacin [2.0 µg/mL]) anti-mycobacterial agents using 7H11 agar. Extended pDST was done on cultures with second-line resistance at critical concentrations using the agar proportion method on 7H11 for BDQ (0.25 µg/mL), CFZ (0.25 µg/mL), LZD (1.0 µg/mL), and MFX (0.5 and 2.0 µg/mL). All isolates whose genomes had an *mmpR5* variant underwent BDQ MIC testing using the agar proportion method on 7H11. Furthermore, isolates with *ddn*, *fgd1*, *fbiA*, *fbiB*, *fbiC*, or *fbiD* variants were tested for DLM MICs using microtiter plates. Isolates were initially cultured in 7H9 liquid medium and adjusted to an optical density of 0.16 at 600 nm. Tenfold serial dilutions were prepared, and 10^−2^ and 10^−4^ suspensions were inoculated onto 7H11 plates containing BDQ concentrations of 0.03, 0.06, 0.12, 0.025, 0.5, 1.0, 2.0, 4.0, and 8.0 µg/mL (15).

#### Drug susceptibility testing using TM4::*GeNL* reporter mycobacteriophage

We performed DST on 15 *Mtb* clinical isolates using the TM4::*GeNL* reporter mycobacteriophage-based assay. All isolates were evaluated for phenotypic resistance to BDQ, PMD, and LZD, and 3 out of 15 isolates had additional DLM phenotypic resistance testing. The TM4::*GeNL* reporter mycobacteriophage was propagated and purified following the methods described previously (28). TM4::*GeNL* DST was performed on cultured isolates in 96-well plates for BDQ, PMD, LZD, and DLM, in technical triplicates, following a standard protocol (28). For each experiment, the *Mtb* H37Rv strain was included as a pan-susceptible control. Drug dilutions were prepared in Middlebrook 7H9 medium supplemented with OADC (10%) but not tyloxapol for each drug, with 50 µL dispensed into each drug treatment well. Middlebrook 7H9 medium with OADC was included as a no-drug control. *Mtb* cells were washed with 7H9 medium without detergents, and cell concentration was adjusted to 10^5^ cells/50 μL based on optical density (OD_600 nm_). A volume of 50 µL of these washed cells was added to both drug-containing wells and control wells. After overnight incubation with drugs at 37°C, the TM4::*GeNL* reporter mycobacteriophage was added to all wells. The following day, luciferase substrate was added to the wells, and the relative luminescence units (RLUs) were measured using a luminescence plate reader (Hidex, Germany). The RLU retention percentage was calculated by comparing the signals from the no-drug control wells with those from the drug-containing wells. A luminescence reduction of ≥95% compared to no-drug control was interpreted as susceptible. Susceptibility, intermediate resistance, and resistance phenotypes were defined as RLU retention of <5%, 5%–10%, and >10%, respectively (28). The RLU percentage cutoff values were defined based on prior optimization and empirical performance of the assay (28). RLU of >100% was possible due to *Mtb* bacilli density and clumping, producing a baseline signal exceeding the no-drug control. This was treated as equivalent to RLU 100%.

#### Whole-genome sequencing and analysis

DNA was extracted from *Mtb*, and the genomic libraries were prepared using NEBNext Ultra II DNA (New England Biolabs, Ipswich, MA, USA) following the manufacturer’s protocol and sequenced on an Illumina NextSeq 500 (San Diego, CA, USA). Adapter trimming was performed with Trim Galore v0.3.7. Reads were mapped to the *Mtb* H37Rv reference genome (NC_000962.3) using BBMap v38.32 with a 98% identity threshold. Duplicates were removed with Picard Tools v2.20, and mean genome coverage was assessed with Qualimap v2.21. Variant calling was conducted with FreeBayes v1.2, requiring ≥4 reads, including ≥1 on each strand, a mapping quality of ≥20, and a base quality of ≥30 (15).

### Statistical methods

The two-sample, unpaired, Mann-Whitney *U* test was used to assess the difference in means between the susceptible and resistant groups. Given the exploratory nature and limited sample size, we did not apply multiple-comparison adjustments and did not treat *P* values as confirmatory.

## RESULTS

The parent PRAXIS study screened 422 patients, of whom 200 DR-TB/HIV patients initiating bedaquiline-containing regimens were enrolled, with 199 available for analysis after exclusion of one ineligible participant (30). From this cohort, serial *Mtb* isolates were available only for a subset of participants with persistently positive cultures and adequate longitudinal sampling. All selected patient isolates had a pre-treatment isolate and at least one subsequent monthly isolate on treatment, with pDST indicating resistance to BDQ, DLM, PMD, or LZD, or WGS with mutations in resistance-associated variants for these drugs. A total of 15 longitudinal clinical isolates were included in this study. We identified six pre-treatment and nine on-treatment clinical isolates from six patients within the PRAXIS study who had treatment-emergent resistance to BDQ, DLM, PMD, or LZD, or pre-treatment BDQ heteroresistance. Of six patients, two had pre-treatment BDQ heteroresistance demonstrated by WGS, two had acquired BDQ resistance, one had acquired multi-drug (BDQ, DLM, and PMD) resistance, and one had an *rrl* mutation with an interim association with LZD resistance but was susceptible by conventional routine DST. Table 1 summarizes clinical characteristics for the six participants in this study, including BDQ adherence, treatment regimens, and treatment outcomes.

**TABLE 1:** Bedaquiline adherence status, treatment regimens, and outcomes for six patients whose *Mtb* clinical isolates (*n* = 15) were tested using the TM4::*GeNL* DST^*c*^

ID	Lineage	Time point	BDQ adherence (%)	Treatment regimen^*a*^	Outcome
P0027	4	BL	n/a^*d*^	PZA, FQ, BDQ, CFZ, ETO, LZD, TRZ, PAS	Treatment failure
		M6	70		
P0082	2	BL	n/a	PZA, FQ, BDQ, CFZ, LZD, TRZ, PAS	Death
		M5	80		
		M6	76		
P0150	4	BL	n/a	PZA, FQ, BDQ, CFZ, LZD, TRZ, PAS, **IMP/CLN**	Death
		M1	100		
		M2	100		
P0200	2	BL	n/a	PZA, EMB, FQ, **BDQ**, CFZ, ETO, LZD, TRZ, PAS	Lost to follow-up
		M6	92		
P3524	2	BL	n/a	PZA, FQ, BDQ, CFZ, ETO, LZD, TRZ, PAS, *DLM*	Death
		M2	100		
		M6	87		
P3532	4	BL	n/a	**INH**, PZA, **EMB**, ETO, FQ, LZD, CFZ, BDQ, *TRZ*, *DLM*	Cured
		M2	n/a^*b*^		

^*a*^ Anti-TB drugs in italics were added during the course of the treatment, while TB drugs in bold were stopped during the treatment duration.

^*b*^ P3532 was in the control arm, and therefore, BDQ adherence was not monitored.

^*c*^ BDQ, bedaquiline; BL, baseline; CFZ, clofazimine; DLM, delamanid; DST, drug susceptibility testing; ETO, ethionamide; IPM/CLN, imipenem/cilastatin; LZD, linezolid; M1, month 1; M2, month 2; M5, month 5; M6, month 6; PAS, para-aminosalicyclic acid; and TRZ, terizidone.

^*d*^ "n/a", not applicable.

### Detection of treatment-emergent bedaquiline resistance

**Fig 1:**
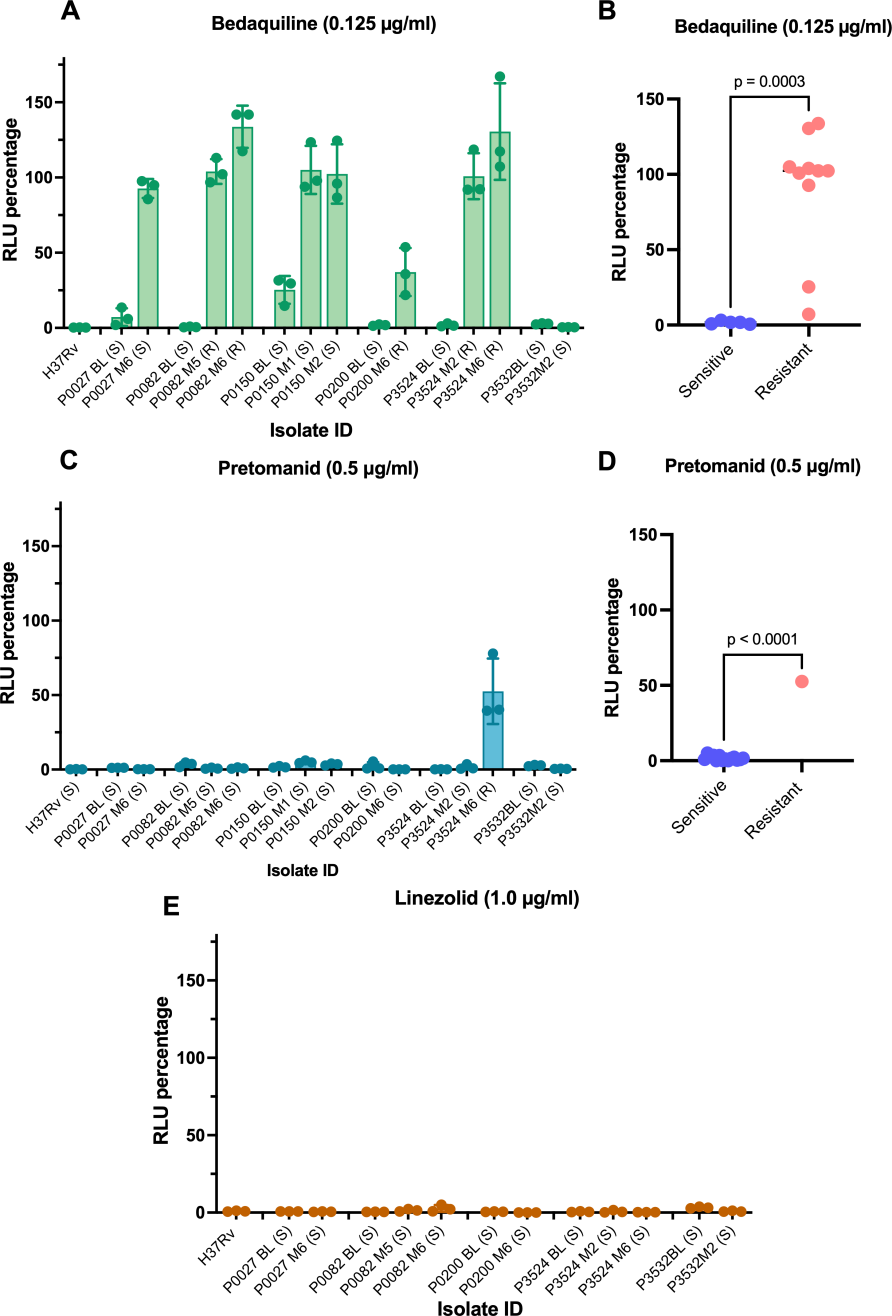
RLU percentage retention of *Mtb* clinical isolates in response to BDQ, PMD, and LZD. (**A**) Detection of on-treatment emerging resistance at BDQ 0.125 μg/mL for three isolates, P0082 at month 5 (M5), P0200 at month 6 (M6), and P3524 at month 2 (M2), by the reporter mycobacteriophage. Baseline isolates (P0027 BL and P0150 BL) demonstrated increased RLU percentage retention, suggesting the presence of BDQ-resistant subpopulations. (**B**) BDQ at 0.125 μg/mL differentiated BDQ drug-resistant and susceptible isolates (*P* = 0.0003). (**C**) RLU percentage retention of *Mtb* clinical isolates in response to PMD at 0.5 μg/mL. P5324 demonstrated on-treatment emerging PMD resistance at month 6 (M6). (**D**) PMD at 0.5 μg/mL differentiated PMD drug-resistant and susceptible isolates (*P* < 0.0001). (**E**) RLU percentage retention of *Mtb* clinical isolates in response to LZD, with all isolates demonstrating LZD susceptible phenotype. Conventional pDST data of each isolate according to the agar proportional method are indicated in parentheses, where (S) and (R) represent susceptible and resistant phenotypes, respectively (**A**, **C**, and **E**). BDQ, bedaquiline; BL, baseline; DST, drug susceptibility testing; LZD, linezolid; M1, month 1; M2, month 2; M5, month 5; M6, month 6; PMD, pretomanid; (S), susceptible; (R), resistant; and RLU, relative luminescence units.

We performed TM4::*GeNL* DST for BDQ on 15 clinical isolates using BDQ 0.125 µg/mL as a concentration that reliably differentiates drug-resistant from susceptible isolates based on previous work (28). This concentration is specific to the reporter mycobacteriophage screening assay and is not interchangeable with the WHO-defined critical concentration (CC) for conventional DST platforms. At 0.125 µg/mL, BDQ-susceptible isolates had luminescence output of 1.5% (95% CI, 0.2–2.8) compared to the no-drug control, while resistant isolates showed a mean luminescence of 83.8% (95% CI, 52.1–115.6) (*P* = 0.0003) (Fig. 1A and B). Similarly, at 0.25 µg/mL, the relative luminescence was retained, and resistance was demonstrated (Fig. S1). The raw RLUs for each drug are shown in Fig. S2.

#### Patient P0082

The baseline sputum culture isolate demonstrated BDQ susceptibility with a mean RLU retention of 0.6%. Subsequent isolates obtained at months 5 and 6 demonstrated a mean RLU retention of 104.0% and 133.7%, respectively, consistent with the emergence of BDQ resistance (Fig. 1A).

WGS data demonstrated the emergence of BDQ resistance-associated variants (RAVs) in the *mmpR5* gene (D47fs [53.3%] and C46fs [35.9%]), which correlated with the resistant phenotype identified by the phage DST (Table 2). Additionally, 7H11 agar-based MICs increased from 0.06 μg/mL at baseline (susceptible) to 1.0 μg/mL, a 16-fold MIC increase at months 5 and 6 (resistant).

**TABLE 2:** Bedaquiline resistance-associated variants identified by whole-genome sequencing, alongside phenotypic bedaquiline susceptibility results for clinical isolates (*n* = 13) tested using the TM4::*GeNL* DST^*a*^

ID	Time point	TM4*::GeNL* Mean RLU% (0.125 μg/mL)	Culture (0.25 µg/mL)	BDQ MIC (μg/mL)	*mmpR5* RAV (%)	WGS mean genome coverage
Emergent bedaquiline resistance						
P0082	BL	0.6	S	0.06	n/a^*b*^	106.6
	M5	104.0	R	1.00	D47fs (53.3) C46fs (35.9)	102.7
	M6	133.7	R	1.00	D47fs (69.8) C46fs (23.1)	94.0
P0200	BL	1.8	S	0.06	n/a	124.4
	M6	102.3	R	0.50	D47fs (92.3)	191.0
P3524	BL	1.8	S	0.03	n/a	215.6
	M2	100.8	R	1.00	D47fs (12.6) Q22P (85.1)	197.3
	M6	130.5	R	0.50	D47fs (45.2) Q22P (9.8) A57E (10.6) R72T (9.2) D88fs (12.0) D88A (13.0) G121R (6.0) L122P (7.4)	165.0
Baseline bedaquiline heteroresistance						
P0027	BL	7.2	S	0.25	R109L (67.9) R156fs (25.8)	82.4
	M6	92.7	S	0.25	R109L (23.9) R156fs (57.1)	117.0
P0150	BL	25.3	S	0.03	E49fs (72.0)	175.5
	M1	105.0	S	ND	E49fs (94.2)	184.4
	M2	102.3	S	0.25	E49fs (96.7)	189.5

^*a*^ MIC, minimum inhibitory concentration; ND, not done; R, resistant; RAV, resistance-associated variant; RLU, relative luminescence unit; S, susceptible. Other abbreviations can be found under Table 1.

^*b*^ n/a, not applicable.

#### Patient P0200

The baseline sputum culture isolate demonstrated BDQ susceptibility, with a mean RLU retention of 1.8%. For the isolate from month 6, the mean RLU retention increased to 37.0%, consistent with phenotypic resistance. These results were concordant with WGS, which demonstrated the emergence of a resistance-associated variant in *mmpR5* (Asp47fs [92.3%]). On 7H11 agar, MICs increased from 0.06 (susceptible) to 0.5 µg/mL, an eightfold MIC increase (resistant) in the corresponding period (Fig. 1A; Table 2).

#### Patient P3524

The baseline sputum culture isolate demonstrated BDQ susceptibility, with a mean RLU retention of 1.8%. The isolates obtained at months 2 and 6 both exhibited a mean RLU retention of ≥100% (Fig. 1A; Table 2). Consistent with RLUs, WGS revealed the emergence of BDQ RAVs in *mmpR5* at month 2 (D47fs [12.6%] and Q22P [85.1%]) and the emergence of further mutations in *mmpR5* by month 6 (A57E [10.6%], R72T [9.2%], D88fs [12.0%], D88A [13.0%], G121R [6.0%], and L122P [7.4%]) (Table 2). On 7H11 agar, MICs increased from 0.03 μg/mL (susceptible) at baseline to 1.0 μg/mL (resistant) at month 2.

### Identifying bedaquiline heteroresistant populations

We identified two patients who had baseline susceptibility to BDQ based on the WHO-defined CC in the 7H11 agar-based method (0.25 μg/mL). For these *Mtb* isolates, WGS identified subpopulations of BDQ RAVs in the *mmpR5*. We tested the baseline and available subsequent isolates for drug resistance using the TM4::*GeNL* reporter mycobacteriophage assay.

#### Patients P0027 and P015

Clinical isolates with baseline *mmpR5* variants but BDQ susceptibility by pDST were selected for further testing with the TM4::*GeNL* assay. We observed RLU percentage retentions of 7.2% and 25.3% at 0.125 μg/mL in the baseline isolates from patients P0027 and P0150, respectively (Fig. 1A; Table 2), suggesting the presence of BDQ-resistant sub-populations. WGS showed *mmpR5* variants (Arg109Leu and Arg156fs) for P0027 BL and Glu49fs for P0150 BL (Table 2). We observed increased BDQ resistance in P0150, where luminescence output increased at 0.125 μg/mL from 25.3% at baseline to >100% at months 1 and 2, with an eightfold increase in conventional phenotypic MIC (7H11 agar) from 0.03 to 0.25 (Table 2).

### Detection of emergent delamanid and pretomanid cross-resistance in patient P3524

Based on previous work, which established 0.5 µg/mL PMD as a concentration that reliably differentiates drug-resistant and susceptible isolates (28), the baseline sputum culture isolate demonstrated PMD susceptibility, with a mean RLU retention of 0.1% at 0.5 µg/mL. At month 6, treatment-emergent PMD resistance was detected, with the isolate demonstrating a mean RLU retention of 52.5% (Fig. 1C and D).

We tested DLM in twofold increasing concentrations between 0.03 and 0.25 μg/mL for all three isolates from patient P3524. This was the only patient who exhibited emerging resistance to PMD. Since cross-resistance between the nitroimidazole class of drugs is common due to the nature of resistance mutations, our objective was to utilize this specific case to demonstrate the reporter mycobacteriophage’s efficiency in detecting cross-resistance between PMD and DLM. At 0.03 and 0.06 μg/mL, a positive control strain *Mtb* H37Rv, P3524 BL, and P3524 M2 maintained a mean RLU of <5%, while P3524 M6 retained a mean RLU of 44.2% and 40.9% at respective concentrations (Fig. S3; Table 3). The RLU retention for P3524 M6 was maintained throughout tested concentrations, with the highest concentration (0.25 μg/mL) demonstrating a mean RLU of 33.8%. WHO recommends a CC of 0.06 g/mL for DLM in mycobacteria growth inhibition tubes (MGIT), and the same CC can be used to define resistance in the reporter mycobacteriophage assay.

**TABLE 3:** Delamanid/pretomanid resistance-associated variants obtained from whole-genome sequencing, conventional phenotypic drug susceptibility testing, and DLM minimum inhibitory concentrations for clinical isolates from patient P3524 tested using the TM4::*GeNL* DST (*n* = 3)^*a*^

ID (lineage)	Time point	TM4*::GeNL* Mean RLU% (0.06 μg/mL)	Culture (0.06 µg/mL)	DLM MIC (μg/mL)	*fbiC* RAV (%)
P3524 (L2)	BL	0.6	ND^*b*^	0.015	n/a^*c*^
	M2	0.4	ND	ND	n/a
	M6	40.9	R	>0.5	A487fs (25.0) S545stop (12.0)

^*a*^ Abbreviations can be found under Tables 1 and 2.

^*b*^ ND, not determined.

^*c*^ n/a, not applicable.

WGS confirmed the emergence of DLM/PMD RAVs in the *fbiC* gene (A487fs, 25.0%; S534stop, 12.0%), correlating with the resistance phenotype identified by the reporter mycobacteriophage DST assay (Table 3). There was an increase in DLM phenotypic MICs from 0.015 μg/mL (susceptible) at baseline to >0.5 µg/mL, a >33-fold MIC increase (resistant) at month 6.

### Detection of emergent linezolid resistance in patient P3532

Similar to the WHO-recommended CC of 1.0 μg/mL LZD, previous work established 1.0 µg/mL LZD as a concentration that reliably differentiates drug-resistant and susceptible isolates using TM4::*GeNL* reporter mycobacteriophage (28). The baseline sputum culture isolate demonstrated LZD susceptibility, with a mean RLU retention of 3.2% (susceptible) at 1.0 μg/mL. At month 2, the isolate demonstrated a mean RLU retention of 0.8% (susceptible) (Fig. 1E).

WGS demonstrated that the isolate obtained at month 2 had a mutation in the *rrl* gene (n.2814G > T, 53.8%). There was no correlation between the genotype observed (suggesting resistance) and phenotype obtained (susceptible) from routine DST and TM4::*GeNL* mycobacteriophage assay. The MICs we performed in MGIT for both isolates confirmed the susceptibility by showing LZD MIC of ≤0.25 µg/mL (Table 4).

**TABLE 4:** Linezolid resistance-associated variant obtained from whole-genome sequencing, conventional phenotypic drug susceptibility testing, and minimum inhibitory concentrations for isolates from patient P3532 tested using the TM4::*GeNL* DST (*n* = 2)^*a*^

ID (lineage)	Sample	TM4*::GeNL* Mean RLU% (1.0 μg/mL)	Culture (1.0 µg/mL)	LZD MIC (μg/mL)	*rrl* RAV (%)
P3532 (L4)	BL	3.2	S	≤0.25	None
	M2	0.8	ND	≤0.25	n.2814G > T (53.8%)

^*a*^ Abbreviations can be found under Tables 1 and 2.

## DISCUSSION

In our study, TM4::*GeNL* reporter mycobacteriophage enabled rapid phenotypic drug susceptibility testing and early detection of treatment-emergent resistance to novel anti-TB drugs, with performance comparable to conventional pDST. The assay identified emergent resistance to BDQ and PMD, key components of modern DR-TB regimens, and detected baseline BDQ resistance missed by conventional testing. Longitudinal increases in reporter signal were concordant with WGS evidence of selection and fixation of *mmpR5* variants, supporting the ability of the TM4::*GeNL* assay to track resistance evolution in real time.

The resistance-associated *mmpR5* variants identified in this study (Asp47fs, Cys46fs, Gln22Pro, and Gly121Arg) have previously been linked to BDQ resistance (15, 31). Frameshift and premature stop codon mutations in *fbiC*, also observed here, are known to confer cross-resistance to PMD and DLM (32). Consistent with this, emergent PMD resistance was detected at month 6 in a patient who initiated DLM at month 1. Although loss-of-function mutations across the nitroimidazole activation pathway (*ddn, fgd1,* and *fbiA-D*) are widely associated with PMD-DLM cross-resistance, exceptions have been reported (23, 33).

One isolate harbored an *rrl* n.2814G > T variant with an interim WHO association with LZD resistance (31); however, both the reporter mycobacteriophage assay and MGIT MIC testing demonstrated a susceptible phenotype. Similar genotype-phenotype discordance has been reported previously, where the isolate with an *rrl* m.2814G > T variant had an MIC of <1.0 µg/mL in MGIT (34). This may reflect the requirement for high allelic frequency or additional contextual factors to meaningfully elevate MICs. Together, these findings highlight challenges in interpreting resistance for drugs with incompletely characterized resistance mechanisms and underscore the need for additional evidence to link specific genotypes to phenotypic resistance.

Bedaquiline heteroresistance due to minor *mmpR5* subpopulations (Arg109Leu and Arg156fs) was not detected by conventional pDST but was identified by WGS and the reporter mycobacteriophage assay, which showed elevated luminescence at the BDQ CC (0.125 µg/mL) in two patients with baseline *mmpR5* variants. Importantly, these variants (Arg156fs and Glu49fs) have been associated with BDQ resistance according to the WHO catalog of mutations (31). Although the MICs remained within the wild-type range, overall susceptibility can mask resistant *Mtb* subpopulations (15). Reduced signal retention observed in heteroresistant isolates likely reflects the coexistence of susceptible and resistant subpopulations, resulting in overlap of RLU% values and limited discrimination from susceptible isolates. This highlights the utility of the reporter phage assay in detecting subtle phenotypic differences, which may be missed by conventional molecular and phenotypic methods. It is also important to select an optimal discriminatory concentration and provide additional supporting results from traditional methods to validate the reporter phage DST findings. We believe that future studies, including a larger number of heteroresistant isolates, will enable refining signal retention cutoffs and improve the accuracy of classification in such cases.

Consistent with prior reports linking *mmpR5* mutations to poor outcomes despite phenotypic susceptibility (16, 19, 35), all five patients in this study with baseline or emergent *mmpR5* variants experienced unfavorable outcomes. These findings underscore the importance of sensitive, rapid DST methods capable of detecting resistant subpopulations and support the reporter mycobacteriophage as a screening tool for emerging resistance to BDQ and other key drugs in modern DR-TB regimens.

The global rollout of BPaL/BPaLM regimens has proceeded despite limited data on resistance to PMD and DLM, largely due to the absence of rapid molecular diagnostics. Current frontline assays detect only known resistance mutations, rendering them inadequate for newer anti-TB drugs, while pDST requires up to 6 weeks for interpretable results. As a result, treatment is often initiated without susceptibility data, risking reduced regimen potency in patients with pre-existing resistance. Against this backdrop, our study provides interim evidence that a reporter mycobacteriophage assay can determine PMD and DLM susceptibility within 48 h of culture positivity, representing a promising rapid alternative that warrants validation in larger cohorts.

Extending previous findings (28–31), we demonstrate treatment-emergent resistance to novel anti-TB drugs using longitudinal clinical isolates, including heteroresistance that is difficult to detect with existing molecular or conventional DST methods. Longitudinal analyses of clinical isolates remain limited in the literature, largely due to challenges in isolate availability. By analyzing serial isolates from patients with persistently positive *Mtb* cultures, we show that reporter mycobacteriophage assays can rapidly identify emerging resistance, particularly for newer anti-TB drugs for which resistance detection tools are limited.

Study strengths include comprehensive characterization of longitudinal isolates using conventional pDST, TM4::*GeNL* DST, and WGS, with concordant discrimination of drug-susceptible and drug-resistant phenotypes across methods. This study also has several limitations: the sample size was small, and no independent validation cohort was included. Serial clinical isolates from patients with emerging resistance are uncommon, and isolates were, therefore, selected for proof-of-concept evaluation. Although longitudinal isolates were extensively characterized using pDST, TM4::*GeNL* DST, and WGS, these findings should be interpreted cautiously. Prospective evaluation in larger, independent cohorts representing diverse DR-TB phenotypes will be necessary to establish diagnostic accuracy and generalizability. Given the slow doubling time of *Mtb* (16–24 h) and the difficulty in achieving complete metabolic inhibition within 48 h, we adopted normalization using mean RLU% retention to improve discrimination between susceptible and resistant isolates. This approach reduces technical variability and captures biologically relevant differences in metabolic activity.

This study supports TM4::*GeNL* DST as a practical alternative to conventional culture-based pDST, particularly in settings where timely resistance detection is critical. The assay provides results within 48 h of culture positivity and enables multiplexed assessment of susceptibility to drugs that form the backbone of contemporary all-oral RR/MDR-TB treatment regimens. This novel assay holds significant promise for low- and middle-income countries, requiring only a luminescence plate reader and minimal expertise for interpretation. By detecting emerging resistance to BDQ and PMD/DLM and BDQ heteroresistance in longitudinal isolates, this approach may facilitate earlier recognition of declining regimen potency and more timely treatment adjustment. Although assay performance depends on sufficient bacterial viability and active metabolism to support phage infection and subsequent luminescence generation via active transcription and translation of the luciferase reporter cassette and appropriate quality controls, these requirements are manageable within routine laboratory workflows. Adequate mycobacterial metabolic activity, maintained by incubation at 37°C, is essential for reliable reporter phage assay performance, as luminescence depends on viable bacteria capable of supporting phage infection and reporter expression. The inclusion of no-drug and quality-control (pan-susceptible) controls enables normalization of signal retention and ensures assay robustness by monitoring bacterial viability and drug potency. Further development to enable direct testing of clinical specimens would enhance clinical applicability. Overall, reporter mycobacteriophage-based DST has the potential to improve resistance detection and treatment outcomes for people with DR-TB.

## SUPPLEMENTAL MATERIAL

Supplemental figures spectrum.03900-25-s0001.docx Fig. S1 to S3.

ASM does not own the copyrights to Supplemental Material that may be linked to, or accessed through, an article. The authors have granted ASM a non-exclusive, world-wide license to publish the Supplemental Material files. Please contact the corresponding author directly for reuse.
