# Supplementary material for: Rapid detection of treatment-emergent resistance to bedaquiline and pretomanid in Mycobacterium tuberculosis using TM4::GeNL reporter mycobacteriophages
Source: Microbiol Spectr. 2026 Jul 15;14(8):e03900-25. doi: 10.1128/spectrum.03900-25 (PMC13436348; doi:10.1128/spectrum.03900-25)
Supplement: Supplementary file 1 [file spectrum.03900-25-s0001.docx]

**Supplementary Figures**

**Supplementary Figure 1**. (A) RLU percentage retention of *Mtb* clinical isolates in response to BDQ at 0.25 μg/ml. RLU is still retained at this concentration demonstrating on-treatment emerging resistance for three isolates, P0082 at month five(M5), P0200 at month 6(M6) and P3524 at month two (M2) by the reporter mycobacteriophage. (B) BDQ at 0.125 μg/ml differentiated BDQ drug-resistant and sensitive isolates (p = 0.0076). Conventional phenotypic DST data of each isolate according to the agar proportional method for BDQ at 0.25 μg/ml is indicated in parentheses, where (S) and (R) represent sensitive and resistant phenotypes, respectively (panel A). **Abbreviation**: BDQ, bedaquiline; BL, baseline; M2, month two; M5, month five; M6, month six; (S), sensitive; (R), resistant; RLU, relative luminescence unit.

**Delamanid µg/ml**

**Supplementary Figure 2**. Raw RLU retention of *Mtb* clinical isolates in response to BDQ, PMD, LZD and DLM. (A) Raw RLU retention in no drug controls and against BDQ at 0.125 μg/ml. (B) Raw RLU retention in no drug controls and against PMD at 0.5 μg/ml. (C) Raw RLU retention in no drug controls and against LZD at 1.0 μg/ml. (D) Raw RLU retention in no drug controls and against DLM at 0.03-0.25 μg/ml. Raw RLU retention appeared more discriminatory between phenotypically resistant and susceptible isolates than mean RLU % retention (Figure 1). Given the slow doubling time of *M. tuberculosis* (16–24 hours) and the difficulty of achieving complete metabolic inhibition and reducing the luminescence retention baseline/noise level within 48 hours, we adopted normalization using mean RLU% retention to improve discrimination between susceptible and resistant isolates. This approach reduces technical variability and captures biologically relevant differences in metabolic activity.

Phenotypic profile of each isolate according to the agar proportional method for BDQ at 0.25 μg/ml and LZD at 1.0 μg/ml, is indicated in brackets, where (S) is sensitive and (R) is resistant (panels A and C). **Abbreviation**: BDQ, bedaquiline; BL, baseline; DLM, delamanid; LZD, linezolid; M1, month one; M2, month two; M5, month five; M6, month six; (S), sensitive; (R), resistant; RLU, relative luminescence unit.

**Supplementary Figure 3**. RLU percentage retention of H37Rv and patient P3524 *Mtb* serial clinical isolates in response to DLM at 0.03 μg/ml to 0.25 μg/ml. Delamanid testing was performed only for patient P3524, as this was the sole isolate in the cohort harboring mutations in one of the six canonical genes associated with nitroimidazole resistance (*ddn, fgd1, fbiA, fbiB, fbiC*, and *fbiD*). Accordingly, DLM MIC testing was not performed for other isolates.

**Abbreviation**: BL, baseline; DLM, delamanid; MIC, minimum inhibitory concentration; M2, month two; M6, month six; RLU, relative luminescence unit.
